# Oxytocin enhances attention to the eye region in rhesus monkeys

**DOI:** 10.3389/fnins.2014.00041

**Published:** 2014-03-03

**Authors:** Olga Dal Monte, Pamela L. Noble, Vincent D. Costa, Bruno B. Averbeck

**Affiliations:** ^1^Laboratory of Neuropsychology, National Institute of Mental Health, National Institutes of HealthBethesda, MD, USA; ^2^Department of Neuropsychology, University of TurinTurin, Italy

**Keywords:** oxytocin, eyes, facial expression, free-viewing, gaze, eye tracking, intranasal oxytocin, rhesus macaques

## Abstract

Human and non-human primates rely on the ability to perceive and interpret facial expressions to guide effective social interactions. The neuropeptide oxytocin (OT) has been shown to have a critical role in the perception of social cues, and in humans to increase the number of saccades to the eye region. To develop a useful primate model for the effects of OT on information processing, we investigated the influence of OT on gaze behavior during face processing in rhesus macaques. Forty-five minutes after a single intranasal dose of either 24IU OT or saline, monkeys completed a free-viewing task during which they viewed pictures of conspecifics displaying one of three facial expressions (neutral, open-mouth threat or bared-teeth) for 5 s. The monkey was free to explore the face on the screen while the pattern of eye movements was recorded. OT did not increase overall fixations to the face compared to saline. Rather, when monkeys freely viewed conspecific faces, OT increased fixations to the eye region relative to the mouth region. This effect of OT was particularly pronounced when face position on the screen was manipulated so that the eye region was not the first facial feature seen by the monkeys. Together these findings are consistent with prior evidence in humans that intranasal administration of OT specifically enhances visual attention to the eye region compared to other informative facial features, thus validating the use of non-human primates to mechanistically explore how OT modulates social information processing and behavior.

## Introduction

There is increasing evidence that the neuropeptide oxytocin (OT), functioning both as a hormone and neurotransmitter, plays a significant role in social behavior across a wide variety of species (Donaldson and Young, 2008; Insel, 2010). A fundamental aspect of effective social interactions in humans and animals is the ability to recognize and interpret facial expressions. This ability can be impaired in several psychiatric disorders, including autism and schizophrenia (Guastella et al., 2010; Averbeck et al., 2011). Studies with autistic patients suggest that intranasal administration of OT improves emotion recognition abilities (Guastella et al., 2010), possibly through increased fixations of the eye region of a face (Andari et al., 2010). Studies in patients with schizophrenia similarly indicate that this neuropeptide improves patients' ability to accurately characterize facial expressions of emotion (Averbeck et al., 2011; Goldman et al., 2011).

The effects of intranasal OT administration in healthy human subjects reinforce clinical evidence that this neuropeptide modulates the ability to recognize, interpret, and infer emotions through visual processing of facial expressions. OT facilitates identity recognition of previously viewed faces (Savaskan et al., 2008) and increases the ability to accurately identify the emotion conveyed by a particular facial expression (Ijzendoorn and Bakermans-Kranenburg, 2012). OT also appears to bias processing of facial valence, based on evidence that OT enhances encoding of happy faces (Guastella et al., 2008b) and decreases aversion to angry faces (Evans et al., 2010). Moreover, OT seems to affect processing of specific facial features. OT administration increases the amount of time people spend fixating on the eyes when they view static pictures of human faces (Guastella et al., 2008a). OT also improves people's ability to recognize others' emotions when these judgments were based on presentations of the eye region of a masked face (Domes et al., 2007). Although much research has led to the common idea of OT as a “pro-social” peptide that improves social behavior and cognition, other studies have suggested a more complex, and not necessarily positive, function for OT (Bosch et al., 2005; Shamay-Tsoory et al., 2009). As in animal studies (Insel and Winslow, 1991), human research has revealed that the effects of OT in the social domain are often weak and inconsistent (Bartz et al., 2011) probably because of the small number of participants, who are often only male, different experimental design, and type of emotional stimuli presented. Conflicting results have been reported about OT effects on recognition of emotional expressions, with some studies reporting effects for fearful expressions (Fischer-Shofty et al., 2010), others only for positive (Marsh et al., 2010) and still others reporting no effect of expressions (Gamer, 2010). Similar inconsistent results have been reported for trusting behavior (Declerck et al., 2010; De Dreu et al., 2010; Mikolajczak et al., 2010) and memory for social stimuli. For example, Savaskan et al. (2008) found that OT improved memory for neutral and angry but not happy faces, whereas Guastella et al. (2008b) found that the effect was only present for happy faces.

To date few studies have investigated the role of exogenous OT in social behavior in non-human primates. Interestingly, it has been shown that when OT is administered intranasally macaques look more often toward other monkeys in the same experimental room (Chang et al., 2012) and at conspecifics' faces in a computer task (Ebitz et al., 2013). Furthermore, in a recent study authors reported that intranasal administered OT suppresses, rather than enhances, species typical vigilance for negative facial expression, but not for neutral or non-social stimuli (Parr et al., 2013).

In this study, we explored whether intranasal administered OT modulates eye movements when macaques view social stimuli. As in humans, face processing in monkeys is an important and rapid process that allows them to identify members of their group, interpret their facial signals, and respond to them with appropriate behaviors (Gothard et al., 2009). We applied a randomized, placebo-controlled, within-subject design to investigate the effects of OT on gaze orienting behavior when monkeys freely view pictures of conspecific faces. Based on previous human studies (Guastella et al., 2008a; Andari et al., 2010; Gamer, 2010) we hypothesized that OT, rather than prompting increased face processing of the entire face, would enhance attention to the eye region when monkeys viewed conspecific faces.

## Materials and methods

### Subjects and experimental setup

Four male adult rhesus monkeys (Macaca mulatta) (6–10 years old, 7–11 kg) B, E, G, and S, served as subjects. All animals were acquired from primate breeding facilities in United States where they had social-group histories as well as group-housing experience until their transfer to NIH for quarantine. After that, they were pair-housed in a rhesus monkey colony room with tactile, auditory, and visual contact with one another. The colony rooms accommodate 24 rhesus monkeys, and the four primates that served as subjects in this study have been housed at NIH between 3 and 4 years prior to this experiment. All subjects therefore have had extensive social experience, thereby making them familiar with perception and interpretation of facial cues in conspecifics. All procedures were performed in accordance with the National Institutes of Health Guide for the Care and Use of Laboratory Animals and were approved by the Animal Care and Use Committee of the National Institute of Mental Health.

Animals had surgically implanted head posts for head fixation to allow for accurate video tracking of eye movements. An Arrington ViewPoint eye tracking system recorded eye movements while monkeys examined each conspecific face. Images were displayed on a computer monitor placed 40 cm in front of the monkey and the face stimuli subtended approximately 13° of visual angle. During the testing phase, all monkeys received controlled access to water.

### Behavioral task

**Figure 1:**
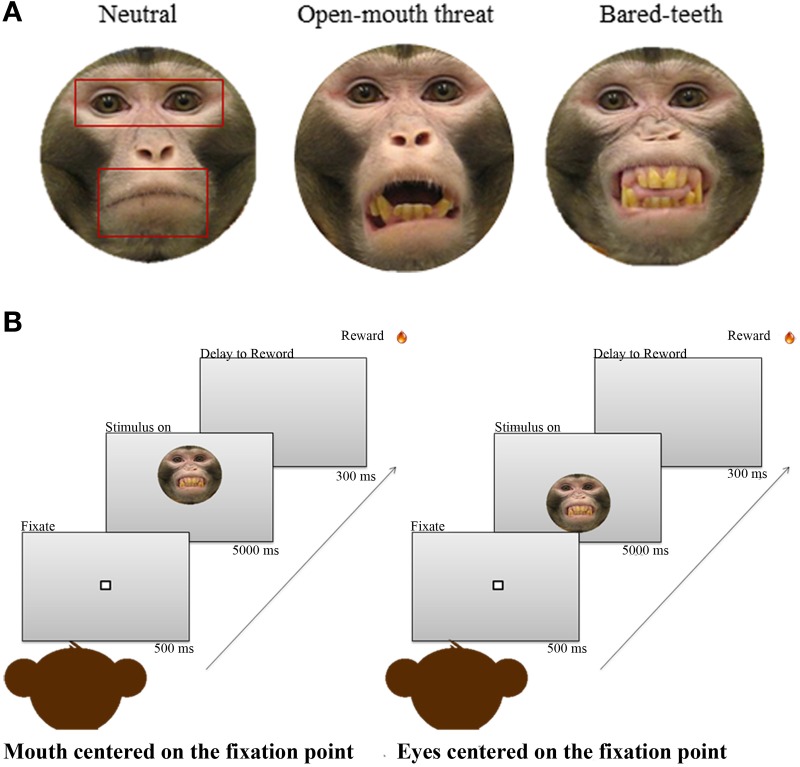
**(A)** One example of each of the three monkey expressions used as stimuli. Superimposed on the neutral expression are the 3 areas of interest (AOIs) used for the analysis; face, eyes, and mouth. **(B)** The free viewing task. The sequence on the left shows a face appearing with the mouth centered at the level of the initial fixation point, and the sequence on the right shows a face appearing with the eyes centered at the level of the initial fixation point.

The task was a free viewing paradigm adopted by previous fMRI studies conducted in humans (Gamer et al., 2010; Kliemann et al., 2012). The monkeys first acquired and held a central fixation point for 500 ms, and then a conspecific image was shown on the screen in front of them for 5 s depicting one of three ecologically relevant facial expressions—neutral, open-mouth threat, or bared-teeth (Figure 1A). During the 5 s period monkeys were free to explore or not each face presented. At the end of the 5 s presentation a juice reward was delivered, regardless of the gaze pattern of the subject. Images were presented randomly in one of two different vertical positions on the screen: either the eyes or the mouth were centered at the level of the fixation point, thus balancing which facial feature was first seen by the monkeys (Figure 1B). The monkeys completed a minimum of 300 valid trials per session and the duration of the session never exceeded 1 h. Valid trials are defined as those in which the monkey successfully fixated on the initial fixation point for 500 ms. If the monkey broke fixation during that required fixation time, the trial was counted as incorrect and no face image appeared. We included all successful trials even if the animals did not look at the regions of interest once the face image appeared.

The set of pictures used were adopted from a recent non-human primate study of fMRI responses to faces (Furl et al., 2012). Subjects were naïve to the free viewing face task, and were unfamiliar with the individual animals whose faces were depicted in the images. All stimuli were color static photographs of a frontal view of a monkey face with direct gaze toward the camera. The set was comprised of 54 different images, with 18 images per expression (neutral, open-mouth threat, or bared-teeth) from three individual adult male monkeys. Neutral expressions show monkeys with a closed mouth. The open-mouth threat expression shows monkeys with an aggressive, threatening facial expression, and bared-teeth fearful expressions display animals with a fearful facial expression (Gothard et al., 2007; Parr et al., 2013). All stimuli were embedded in a gray oval mask as background.

Although we repeatedly presented the same set of stimuli, the total number of fixations on the face region did not significantly differ within session [*F*_(1, 50)_ = 0.81 *p* = 0.37] or across sessions [*F*_(1, 40)_ = 1.48, *p* = 0.23). Additionally, as well as the total number of fixations, the total looking time at the face region did not significantly differ within session [*F*_(1, 39)_ = 0.07 *p* = 0.79] or across sessions [*F*_(1, 40)_ = 1.75, *p* = 0.19]. Furthermore, we explored whether there was an effect of OT on habituation to images within a session. We did not find any significant effect of drug by number of images repetition for total number of fixations on the face region [*F*_(1, 50)_ = 0.34 *p* = 0.56] or for total looking [*F*_(1, 39)_ = 0.06 *p* = 0.94].

### Intranasal OT administration

Prior to beginning the experiment the monkeys were habituated to receiving saline nasal spray. During each puff in one nostril the other nostril and the mouth were gently held closed, thus encouraging the animal to inhale the spray. The animals' heads were fixed for this procedure, to minimize movement and enhance the reliability of dosing. This habituation procedure was repeated until the monkeys were completely relaxed during the nasal spray administration.

On the day of the experiment monkeys were transported in a primate chair from the colony room to the experimental room. After fixing their heads, intranasal doses of 24 IU OT (Sigma) or sterile saline were given in a 1 mL volume. This is similar to the dose previously found to affect socially relevant behaviors in monkeys (Chang et al., 2011) and humans (Kirsch et al., 2005; Guastella et al., 2008b; Rimmele et al., 2009; Evans et al., 2010). Behavioral testing began 45 min after each treatment. It has been shown that vasopressin, which is closely related to OT, reaches peak levels in CSF in 30–50 min when administered to humans intranasally (Born et al., 2002) and a 45 min delay between drug administration and the start of testing was used in previous human studies (Guastella et al., 2009). As in other pharmacological studies with non-human primates (Chang et al., 2012; Feifel et al., 2012; Ebitz et al., 2013) we did not use a double-blind design; as the data collection is automatically recorded using the eye tracking system, any possible researcher bias should not influence results. Doses of saline and OT were balanced across sessions and were administered on alternating days (Chang et al., 2012; Ebitz et al., 2013) at least 5 sessions of OT and 5 of saline were collected for each animal. There were a total of 25 OT sessions (number of session for each monkey: 7, 6, 7, 5) and 21(number of session for each monkey: 5, 5, 6, 5) saline sessions included in the analysis.

### Data analysis

The number of fixations was defined for three areas of interest (AOIs): one placed around the whole face, one placed around the eyes, and one placed around the mouth (Figure 1A). The mouth and eye AOIs were equivalent in total area, but differed in shape to accommodate differences in facial features, and the size of the regions were the same across all expressions. We delineated AOIs to quantify the amount of attention the monkeys directed toward the whole face and for specific facial features (eyes and mouth). The AOI around the face was used to investigate if OT increased interest in looking at a face in general, and the AOIs inside the face (eyes and mouth) were for discriminating whether fixations differed between the two regions. For each animal the total number of fixations was calculated using MATLAB (Math Works, Inc., Natick, MA, USA) custom designed programs that calculated all the points that fell within the boundaries of the three AOIs. A fixation and its location were defined as the mean coordinates corresponding to the period of time between successive saccades. Saccades were found by locating points of negative going acceleration zero-crossings that also exceeded a speed threshold in the eye movement data. These points correspond to maxima in the speed profile and mark the midpoints of saccades. The speed threshold insured that random fluctuations and noise were not detected as saccades. After the speed maximum was identified, the algorithm searched forwards and backwards until the speed fell below a pre-specified threshold. These points were then marked as the beginning and end of the saccade (Averbeck et al., 2003).

We normalized data within trials to control for individual differences and variations in number of fixations across test days (Ebitz et al., 2013). The proportion of fixations made within the face region was normalized by dividing by the total number of fixations made outside the face region on each trial. The proportion of fixations made within the eye or mouth regions were both normalized by the total number of fixations made within the entire face region on each trial. All analyses were computed using normalized data.

First we examined whether OT affected the proportion of fixations (dependent variable) made within the face region via a mixed-effect ANOVA that specified drug (OT/saline), initial face position (eyes centered/mouth centered), and facial expression (neutral, open-mouth threat, or bared-teeth) as fixed factors and session number (46; 25OT and 21 Saline) as a random effect nested under monkey (4 subjects) and crossed with drug (OT/saline).

Second, we investigated whether OT affected the proportion of fixations (dependent variable) in the two face region AOIs: eyes and mouth. For the dependent measure we calculated a mixed-effects ANOVA model specifying drug (OT/saline), face position (eyes centered/mouth centered), facial expression (neutral, open-mouth threat, or bared-teeth), and regions (eyes and mouth AOI) as within-subject factors and session number (46; 25OT and 21 Saline) as a random effect nested under monkey (4 subjects) and crossed with drug (OT/saline). Direct *post-hoc* comparisons were made with two-tailed independent *t*-tests and the *p*-value was Bonferroni corrected for the number of comparisons.

Finally, to further investigate the time looking in each AOI we run the same two ANOVA models (one for the face and one for the eyes and mouth AOI) with proportion of time looking as dependent variable. As for the number of fixations we normalized the time within trials. The proportion of time spent within the face region was normalized by dividing by 5 s (time that the conspecific picture is displayed on the screen). The proportion of time spent within the eye and mouth regions were both normalized by the total looking time made within the entire face region on each trial. We also investigate the correlation between proportion of fixations and proportion of time in each AOI.

## Results

### Face processing

We began by examining if OT, facial expression, and initial face position influenced how often the monkeys fixated on the presented face. Neither the drug administered [*F*_(1, 42)_ = 0.3, *p* = 0.58] or the facial expression shown [*F*_(2, 42)_ = 0.2, *p* = 0.84; Figure 3A] or initial face position [*F*_(1, 42)_ = 1.8, *p* = 0.19] affected the relative proportion of fixations to the face, and there was no evidence of a higher order interaction involving either factor (all *p* > 0.05).

### Eye and mouth region processing

Next we examined how often the monkeys fixated on the eyes or mouth based on the defined AOIs (Figure 1A). The monkeys fixated the eyes more than they fixated the mouth region [Region, *F*_(1, 43)_ = 166, *p* < 0.001; Figure 3B]. This preference was modulated by which facial feature was centered at the initial fixation point [Region × Initial Face Position, *F*_(1, 89)_ = 94, *p* < 0.001]. When the eye region was centered on the initial fixation point the proportion of fixations in the eye region increased, compared to when the mouth region was presented at the central fixation point [*t*_(89)_ = 24.4, *p* < 0.001]. Likewise the proportion of fixations in the mouth region was larger when it was centered on the initial fixation point compared to the eye [*t*_(89)_ = −17.1, *p* < 0.001].

**Figure 2:**
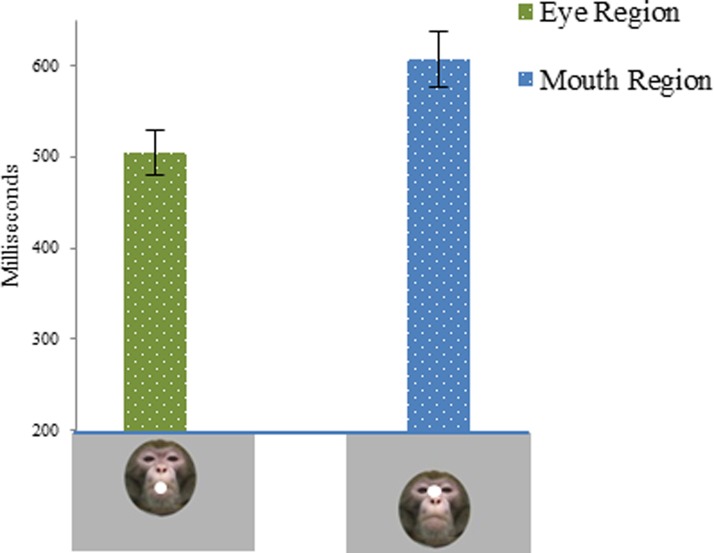
**Latency of the first saccade into the AOI opposite the initial fixation point expressed in milliseconds, as a function of the initial face position.** First column shows latency of the first saccade to the eye region when the face was mouth centered. The second column shows latency of the first saccade to the mouth region when the face was eye centered.

We further investigated the latency of the first saccade into the ROI opposite the initial fixation point (Figure 2). The initial face position affected the latency of the first saccade to each region [Initial Face Position × Region, *F*_(1, 25)_ = 12.44, *p* < 0.001]. When the initial fixation point was located at the mouth region, the latency of the first saccade to the eye region was significantly shorter than the latency of the first saccade to the mouth region [*t*_(44)_ = −4.66, *p* < 0.001] when the initial fixation point was located at the eye region. However, there were no significant effects of drug on the latency of the first saccade to each region. Moreover, we investigated the direction of the first saccade. When the face was mouth centered the 42% of the first saccades were directed on the eye region, whereas when the face was eye centered the 30% of the first saccades landed on the mouth region.

### OT effects on the eye and mouth region

**Figure 3:**
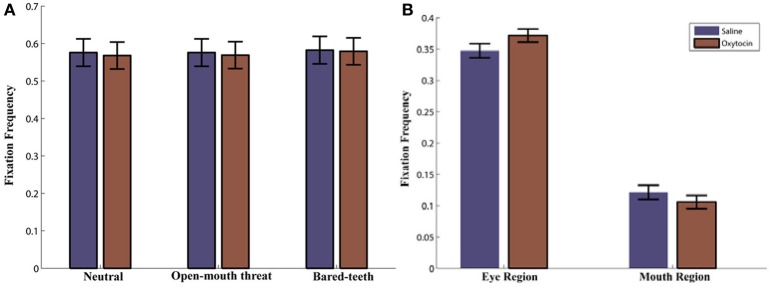
**(A)** Proportion of Fixations to the Face AOI as a function of drug (oxytocin vs. saline) and expression (neutral, open-mouth threat, or bared-teeth). Note that this is averaged over the initial face position. **(B)** Proportion of Fixations to the eye and mouth AOI as a function of drug (oxytocin vs. saline). Note that this is averaged over face expressions.

**Figure 4:**
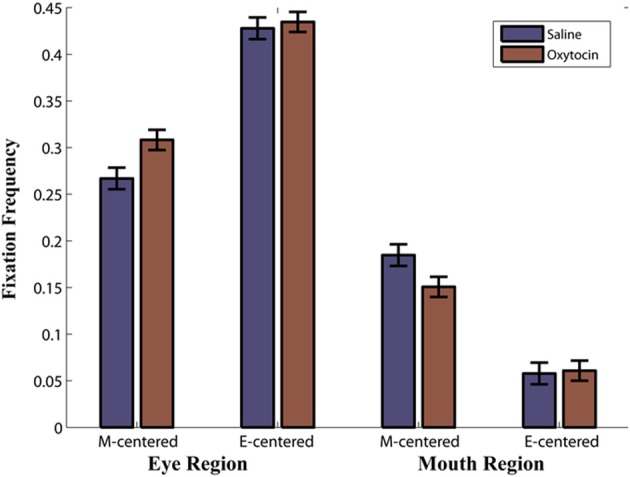
**Proportion of Fixations as a function of drug (oxytocin vs. saline) by region (eye region vs. mouth region) by initial face position (mouth centered vs. eyes centered).** For example, M-centered represents a face that appeared with the mouth centered on the initial fixation point and E-centered represents a face that appeared with the eyes centered on the initial fixation point. Note that this is averaged over expressions.

**Figure 5:**
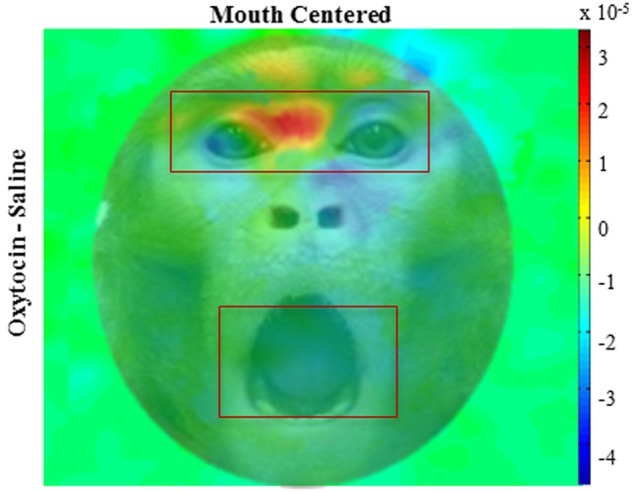
**Fixation density plots.** Colors indicate normalized fixation density as a function of drug (oxytocin minus saline) and initial face position (mouth centered); overlaid on example monkey face. The panel illustrates pattern of eye movement where red indicates more fixations, blue indicates less. All plots are averages per monkey across expressions.

We compared the effects of OT on fixations to the eyes vs. the mouth, and found that OT enhanced the general tendency of the monkeys to fixate on the eyes relative to the mouth [Drug × Region, *F*_(1, 113)_ = 6.2, *p* = 0.02; Figure 3B). Specifically, the difference in how often the monkeys fixated on the eyes vs. the mouth was heightened on OT compared to saline. While OT caused a significant increase in fixations to the eye region, this effect varied based on whether the eye or mouth region overlapped the central fixation point [Drug × Region × Initial Face Position, *F*_(1, 68)_ = 6.5, *p* = 0.01; Figure 4). When the eye region was centrally presented, OT had no impact on fixations to the eye [*t*_(68)_ = −0.2, *p* > 0.05] or the mouth region [*t*_(68)_ = −0.1, *p* > 0.05]. However, when the mouth region was centrally presented, OT compared to saline caused a proportional increase in how often the eye region was fixated [*t*_(68)_ = 2.1, *p* = 0.03], and a parallel decrease in how often the mouth region was fixated [*t*_(68)_ = −2.7, *p* = 0.008]. Thus, OT specifically enhanced scanning of the eye region when that facial feature was not centrally presented. To illustrate the effect of OT on gaze orienting behavior we plotted patterns of fixation density when the mouth region was centrally presented (Figure 5) as a function of drug condition (OT minus saline).

When we carried out analyses on the proportion of time spent in each AOI, all statistics were consistent. In other words, significant effects reported on proportion of fixations were still significant, and non-significant effects were still non-significant. Additionally, proportion of time spent viewing the face, eye, or mouth region was respectively correlated with the proportion of fixations made to each region [Face region *r* = 0.754 *p* < 0.001; Eye region *r* = 0.930 *p* < 0.001; Mouth region *r* = 0.904 *p* < 0.001].

## Discussion

The goal of our study was to examine the effects of OT on gaze behavior in macaques during face processing. We hypothesized that OT might not generally increase social attention, but instead bias social attention uniquely toward the eye region of another monkey face. This would replicate similar findings in humans (Guastella et al., 2008a; Andari et al., 2010; Gamer, 2010) and warrant future mechanistic studies in non-human primates to understand how OT influences social processing. Results indicated that OT did not broadly enhance face processing during free viewing of conspecific faces. Instead, OT increased the relative number of fixations made to the eye vs. mouth region. This implies that OT increases selective attention to the eye region of the face. Interestingly, this effect of OT was most pronounced when the position of the face on the screen was manipulated so that the eye region was not the first facial feature seen by the monkeys.

The first aim of the current study was to investigate the effects of OT on gaze orienting behavior when monkeys viewed pictures of unfamiliar conspecifics' faces. We found that OT did not increase the proportion of fixations to the face, compared to saline. This appears to contrast with a recent study that also investigated OT effects in macaques during an unconstrained viewing task (Ebitz et al., 2013). In that study, monkeys viewed two pictures at a time positioned on either side of an initial fixation point, and the authors reported that OT increased the total time that the monkeys looked at both images. Different results could be due to differences in the task, location of initial fixation point in relation to where the images were presented, stimuli used, as well as data analysis techniques. Ebitz and colleagues showed only familiar (cage-mate) faces with neutral expressions and allowed the monkeys to view the faces until they stopped looking at the images for at least 500 ms. By comparison we had monkeys view three unfamiliar facial identities portraying three different expressions, displayed one at a time, and the monkeys were free to view or not view each face for up to 5 s. However, emerging studies have started to support the idea that OT has a strong effect on the earliest stage of social information processing (Domes et al., 2010; Gamer, 2010; Gamer et al., 2010; Ellenbogen et al., 2012; Ebitz et al., 2013; Parr et al., 2013), and the lack of significant OT effects on overall fixations to the face reported in our study could be the result of an extended face presentation period. Furthermore, we found that the effects of OT were independent of the emotional expression presented. Conflicting results have been reported about OT effects on emotional expressions (Fischer-Shofty et al., 2010; Gamer, 2010; Marsh et al., 2010) and more work is needed investigate if OT affects particular expression types and to clarify the different results reported in literature.

Independently from the OT manipulation, we found that the monkeys preferred to fixate on the eyes relative to the mouth. This is consistent with prior evidence that monkeys made more saccades to the eyes than any other facial feature (Nahm et al., 1997). Keating and Keating (1982), who were among the first researchers to study how monkeys explore facial expressions in a laboratory setting, found that the eye region was a strong attractor of fixations compared to other parts of a face. In our study we used high-resolution images providing details not only of the eyes but also others features of the face (i.e., mouth and teeth). In rhesus monkeys, the expressive differences in the eye region are less dramatic than those in the mouth region (monkeys have very large teeth, and display them prominently in some expressions) making the mouth an overtly informative region to explore. The tendency of the monkeys to explore the eye over the mouth region is also supported when we investigate the direction and the latency of the first saccade as a function of drug and initial face position. We found that when the mouth was the first region presented, the first saccade was more often and faster to the eye region compared when the first feature presented was the eye region. There were not significant effects of drug, suggesting that the importance of the eye region in gleaning socially relevant information may override OT. This is similar to what has been seen in human participants (Enticott et al., 2012) where changes in eye expression play a critical role in effective social communication. The eyes capture significantly more attention than do other parts of the face both in adults (Janik et al., 1978), and infants (Farroni et al., 2002) and participants are equally capable of recognizing specific emotions when they are shown just the eye region or an entire face (Baron-Cohen et al., 1997).

When we examined how OT affected fixations to the eye and mouth regions we found that OT heightened attention to the eye region when monkeys' viewed conspecific faces, relative to saline. These findings concur with prior studies examining how OT influences processing of the eyes in both monkeys (Ebitz et al., 2013) and humans (Guastella et al., 2008a; Andari et al., 2010; Gamer, 2010). Guastella et al. (2008a) tested whether OT increased gaze toward the eye region when viewing neutral faces. A single dose of intranasal OT increased the number and duration of fixations made to the eye region of a face. Additionally, Gamer et al. (2010) found that OT increased the likelihood of gaze changes toward the eyes. Critical information is taken from the eyes (Haxby et al., 2002), and the amount of fixation to the eyes has been found to be predictive of one's ability to interpret the intentions of others and the meaning of social situations (Garrett et al., 1997; Klin et al., 2002; Spezio et al., 2007). Enhanced fixation to the eye region independent of a conspecific's facial expression may be one of the mechanisms underlying the positive effects of OT on facial processing and emotion recognition.

The critical OT effect on the eye region of a face was confirmed and emphasized when we analyzed the proportion of fixations in the eye and mouth region as a function of drug and initial face position. In our experiment we systematically manipulated the vertical position of the presented face so that the initial gaze of the monkey was centered on either the mouth or eye region, which prevented the monkeys from covertly deploying attention to a specific facial feature. Consistent with effects seen in humans (Challinor et al., 1994; Gamer et al., 2010; Kliemann et al., 2010, 2012; Arizpe et al., 2012), our findings indicated that varying the presentation location of the stimuli affects patterns of eye movements. Despite the general preference of the monkeys to explore the eye region, both the eyes and mouth were fixated more often when that particular region was centered over the initial fixation point. Using the same manipulation of initial face position as the current study, intranasal OT is found in humans to refocus attention to the eye region when another facial feature was seen first (Gamer, 2010). The present results indicate this is also the case for rhesus monkeys. When the mouth region overlapped with the initial fixation point, OT caused monkeys to look more often at the eye region and less at the mouth than they did on saline. By contrast when the eye region was centered on the initial fixation point OT had no effect; possibly because the monkeys were already in a position to explore the most informative and interesting feature of a face.

Several limitations to this study should be noted. A sample size of four monkeys may seem small compared to human studies that have investigated behavioral effects of intranasal OT, although it is consistent with typical sample sizes used in psychopharmacological studies involving non-human primates (Chang et al., 2012; Ebitz et al., 2013). An advantage to using non-human primates is that subjects can be brought back repeatedly to determine the consistency of drug related effects across repeated sessions within individual animals. Another point is the lack of a non-social stimulus as a possible control for the effects of OT on overall fixations, which may be independent of the social relevance of the image being viewed. Moreover, we only tested male monkeys so we cannot assume that OT in female yields similar findings. Eye-tracking studies with human male participants have shown that OT increases gaze time spent exploring the eye region compared with other parts of a face (Guastella et al., 2008a; Andari et al., 2010; Gamer, 2010), but two other studies, however, have not replicated this finding in female participants (Domes et al., 2010; Lischke et al., 2012a). Additionally, in males, OT tends to elicit decreased amygdala activity in response to emotional faces (Domes et al., 2007); in females, OT enhances reactivity to social and non-social threat (Domes et al., 2010; Lischke et al., 2012b). Future studies should include both sexes to determine the behavioral, neural, and physiological effects of OT on gender differences in order to make progress in understanding the function and potential utility of OT in treatment.

Finally, behavioral effects that follow peripheral administration of OT could be driven by at least three mechanisms. First, the peripherally administered OT could enter the CNS and bind to OT receptors there. Second, the peripherally administered OT may drive elevation of CNS OT via an unknown, indirect peripheral mechanism. In this case, OT binding to peripheral OT receptors may be driving changes in central OT levels. Finally, the peripherally administered OT may lead to behavioral effects via an entirely peripheral mechanism. There are many OT receptors in several peripheral structures including kidneys and pancreas, as well as in the heart, fat cells, and adrenal glands (Gimple and Farenholtz, 2001). Which of these three mechanisms is giving rise to the behavioral effects is not currently known. In addition, different intranasal delivery methods may also operate through any of these three mechanisms, and the mechanism engaged by any delivery method may vary among species. At present, little is known about this and more research will be necessary to clarify these questions.

In summary, intranasal administered OT in rhesus monkeys did not increase overall interest in exploring conspecifics' faces compared to saline. Instead, OT increased the number of fixations made to the eye region when the animals were allowed to freely explore monkey faces. Further, when the vertical position of the presented face was shifted to control for which feature was seen first, OT specifically enhanced attention to the eye region. Together these findings are consistent with prior evidence in humans and non-human primates that intranasal administration of OT specifically enhances social attention to the eye region compared to other informative facial features. We conclude that this supports the utility of a primate model in investigating the neurobiological mechanisms involved in the perception and processing of social information, and the role OT plays in those processes.

## Author contributions

Bruno B. Averbeck designed research; Olga Dal Monte and Pamela L. Noble performed research; Olga Dal Monte and Vincent D. Costa. analyzed data; Olga Dal Monte, Pamela L. Noble, Vincent D. Costa, and Bruno B. Averbeck wrote the paper.

### Conflict of interest statement

The authors declare that the research was conducted in the absence of any commercial or financial relationships that could be construed as a potential conflict of interest.
