# Supplementary material for: Oxytocin enhances attention to the eye region in rhesus monkeys
Source: Front Neurosci. 2014 Mar 3;8:41. doi: 10.3389/fnins.2014.00041 (PMC3939446; doi:10.3389/fnins.2014.00041)

Figures and Plots for Reviewers:

- Even if the effect size remains the same, it is worth further exploring these data. How does oxytocin affect the total looking time allocated to each facial region? What about the duration of fixations on the eyes?

Below we plotted for the reviewer proportion of time and proportion of fixations for the eye and mouth regions to show that time and fixations have a very similar pattern.

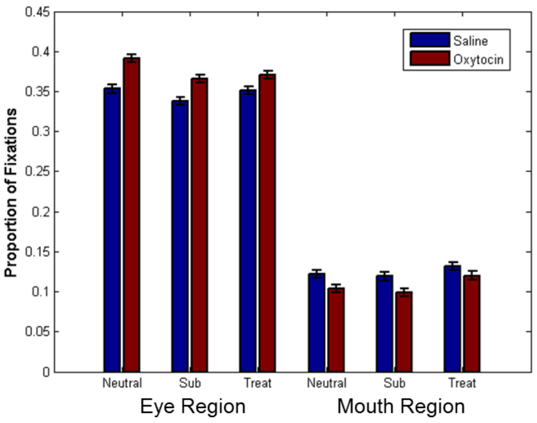

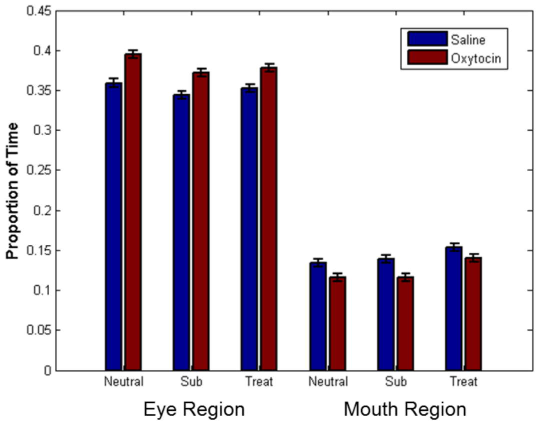

- Is there a difference in the time spent fixating the eyes early vs. late in the 5 s period?

Reply: To address this we calculated how many saccades animals made in each trial and we plotted the proportion of fixations for each ROI (eye and mouth) accounting for drug condition and initial face position (eye and mouth centered). On the Y axis the proportion of fixations and on the X axis the number of saccades per trial. Below the 2 plots for the 2 region of interests.

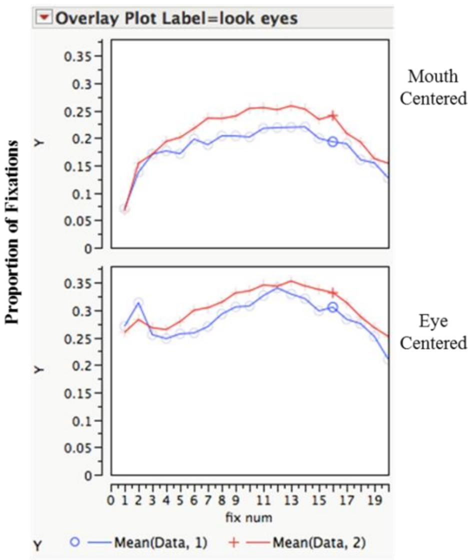

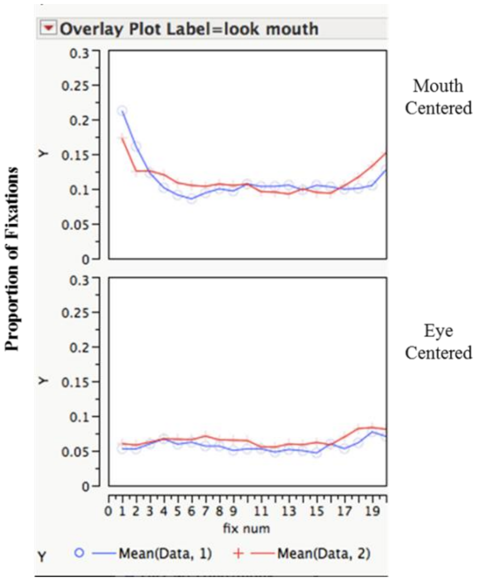

- (2) With the current method of data analysis, the scanpath is quantified by counting the number of fixations within a region and normalizing this number relative to the total number of fixations. This normalization procedure aids in some aspects of the data analysis but also leads to some confusing interpretations of the data. For example, if the monkey only makes 1 fixation on the face and that fixation is on the eyes, the normalization ratio is 1. However, if he makes 10 fixations on the face and, again all the fixations are on the eyes, the ratio is still 1. Although these two looking-behaviors are quite different, normalizing the data has the effect of reducing them to quantitatively the same behavior.

Here we show two plots of fixation inside the face region (Oxytocin in red and Saline in blue). The first one shows the proportion of fixations made within the face region dividing by the total number of fixations made outside the face region on each trial. The second plot shows the number of fixation within the face region without accounting for the total number of fixations made in each trial.

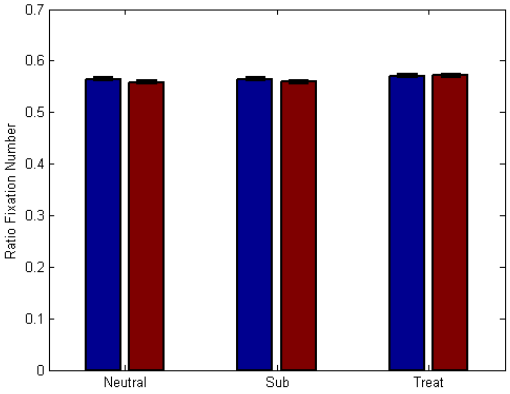

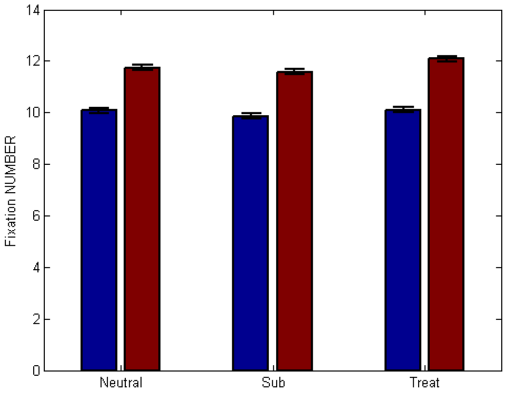

- (2) Does oxytocin influence the behavior of every subject monkey? Please report individual data.

Reply: Below we reported the individual data from the 4 monkeys tested. The 2 heat maps depict Oxytocin minus Saline averaged across expressions. Panel A indicates fixation density plots for images presented with the eye centered at the initial fixation point and panel B for mouth centered. B, G, E, and S are the individual subjects.

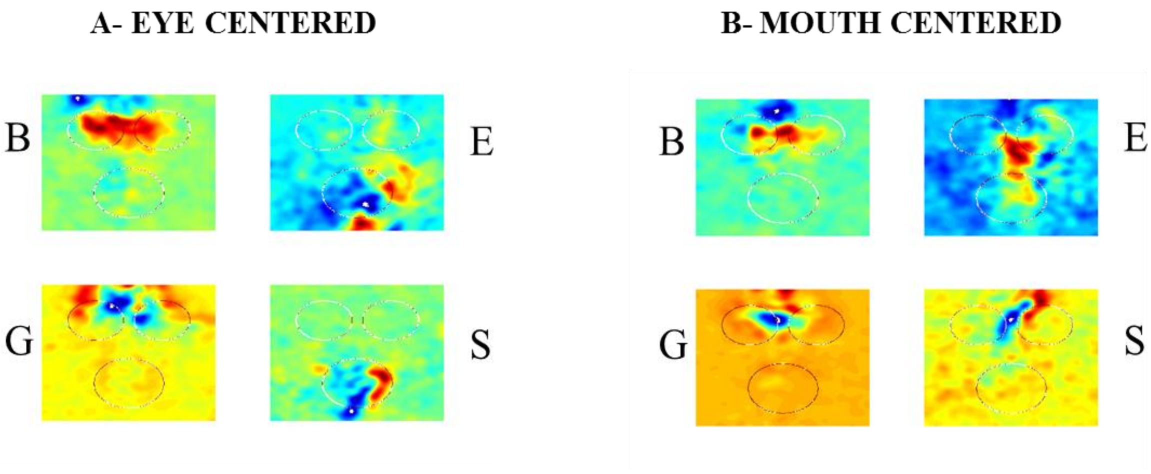

Supplement: Supplementary file 1 [file DataSheet1.PDF]
